# Iron, Copper, and Magnesium Concentration in Hair and Risk of Esophageal Cancer: A Nested Case-Control Study

**DOI:** 10.34172/aim.2023.98

**Published:** 2023-12-01

**Authors:** Maryam Hashemian, Hossein Poustchi, Maryam Sharafkhah, Akram Pourshams, Fatemeh Mohammadi-Nasrabadi, Azita Hekmatdoost, Reza Malekzadeh

**Affiliations:** ^1^Digestive Oncology Research Center, Digestive Diseases Research Institute, Tehran University of Medical Sciences, Tehran, Iran; ^2^Biology Department, School of Arts and Sciences, Utica University, Utica, NY, USA; ^3^Liver and Pancreatobiliary Diseases Research Center, Digestive Diseases Research Institute, Tehran University of Medical Sciences, Tehran, Iran; ^4^Food and Nutrition Policy and Planning Research Department, National Nutrition and Food Technology Research Institute (NNFTRI), Faculty of Nutrition and Food Technology, Shahid Beheshti University of Medical Sciences, Tehran, Iran; ^5^Departments of Clinical Nutrition and Dietetics, Faculty of Nutrition and Food Technology, National Nutrition and Food Technology Research Institute, Shahid Beheshti University of Medical Sciences, Tehran, Iran

**Keywords:** Cancer, Copper, Esophageal squamous cell carcinoma, Iron, Magnesium, Minerals

## Abstract

**Background:**

An association has already been hypothesized between iron, copper, and magnesium status assessed through food frequency questionnaires (FFQs) and the risk of esophageal squamous cell carcinoma (ESCC). However, self-reported dietary assessment methods are prone to measurement errors. We studied the association between iron, copper, and magnesium status and ESCC risk, using hair samples as a long exposure biomarker.

**Methods:**

We designed a nested case-control study within the Golestan Cohort Study, that recruited about 50000 participants in 2004-2008, and collected biospecimens at baseline. We identified 96 incident cases of ESCC with available hair samples. They were age-matched with cancer-free controls from the cohort. We used inductively coupled plasma mass spectrometry (ICP-MS) to measure iron, copper, and magnesium concentrations in hair samples. We used multiple logistic regression models to determine odds ratios and 95% confidence intervals.

**Results:**

Median concentrations of iron, copper, and magnesium were 35.4, 19.3, and 41.7 ppm in cases and 25.8, 18.3, and 50.0 ppm in controls, respectively. Iron was significantly associated with the risk of ESCC in continuous analysis (OR=1.41, 95% CI=1.03-1.92), but not in the tertiles analyses (OR_T3 vs. T1_=1.81, 95% CI=0.77-4.28). No associations were observed between copper and magnesium and ESCC risk, in either the tertiles models or the continuous estimate (copper: OR_T3 vs. T1_=2.56, 95% CI=1.00-6.54; magnesium: OR_T3 vs. T1_=0.75, 95% CI=0.32-1.78).

**Conclusion:**

Higher iron status may be related to a higher risk of ESCC in this population.

## Introduction

Esophageal cancer continues to be a significant contributor to global cancer mortality.^1^ Substantial variations in age-adjusted incidence, mortality, and disability-adjusted life-year rates persist among countries, highlighting a primary epidemiological characteristic of esophageal cancer.^1^ Previous studies have proposed minerals to be related to esophageal cancer^2-4^ and its variation.^5^ Iron can produce reactive oxygen species, which can damage DNA and cause gene mutation.^6^ Intracellular iron modulates the signaling pathways in colon cancer,^7^ and the MEK/ERK pathway in squamous cell carcinoma of the head and neck.^8^ Previous studies demonstrated that iron may be related to esophageal carcinogenesis. Keszei et al showed that in the Netherlands Cohort Study, high consumption of heme iron was related to increased risk of esophageal squamous cell carcinoma (ESCC).^9^ Conversely, Cross et al reported that in the U.S. NIH-AARP Study, dietary heme iron intake was not related to the risk of ESCC.^10^ Subclinical magnesium deficiency has been shown to enhance the risk of ESCC.^11^ Copper is related to colon cancer and other types of cancer.^12^

Previous studies on mineral status and ESCC have used data from food frequency questionnaires (FFQs). Dietary intake data has some limitations; for example, it does not account for individual differences in mineral bioavailability and absorption.^13^ Therefore, using a biomarker can provide us with a better estimation of mineral status.^14^ The mineral composition of hair and nails reflects a stable and longer-term estimate of mineral status relative to blood and urine mineral concentration.^15^ In our previous study, we used toenail samples to investigate the relationship between mineral status and the risk of ESCC.^16^ However, due to terrestrial contamination of nail samples, we could not assess the relationship between all minerals and the risk of ESCC.^17^

 This study was designed to evaluate the relationship between iron, copper, and magnesium status in hair and the risk of esophageal cancer in the Golestan Cohort Study. Golestan is a region with a high prevalence of esophageal cancer.

## Materials and Methods

###  Participants

From 2004 to 2008, the Golestan Cohort study recruited about 50 000 subjects older than 40 years in Golestan, Iran, and collected biospecimens including hair samples at baseline. The details of the protocol have been reported before.^18,19^ Briefly, trained technicians interviewed participants and collected information on demographic characteristics and dietary intake. An FFQ was developed and validated for this population.^20-22^ Weights and heights were also measured.^23^

The method for ascertainment of esophageal cancer has been reported previously.^18^ In brief, all subjects were contacted by phone annually to record any hospitalization or disease. Participants who reported GI endoscopy or cancer diagnosis were visited at home by a team and contacted by the clinic. The Digestive Diseases Research Institute of Tehran University reviewed and verified upper GI cancer reports and compared them with the Golestan Cancer Registry. Loss to follow-up was minimal ( < 1%).

 We designed a nested case-control study. For each case, we selected one control randomly without replacement from the participants who provided a hair sample at baseline and were cancer-free at the time the case occurred (a risk set sampling also called concurrent sampling). Controls were matched with cases in terms of age (within one year). Incident cases and controls were identified between January 1, 2004 and June 1, 2014 in the GCS.

###  Ascertainment of Hair Mineral Concentrations

At cohort entry, 1 × 1-inch hair samples were collected from the occipital area of most participants’ heads. Sealed paper bags were used to store hair samples at room temperature. Mineral concentrations were assessed by inductively coupled plasma mass spectrometry (ICP-MS). ICP is utilized to ionize the atoms within the sample, which are then identified according to their mass-to-charge ratio. The high level of accuracy, precision in measurements, and minimal interference make ICP-MS one of the most valuable techniques for trace analysis of biomedical samples. The intra-assay coefficients of variability (CV) for iron, magnesium, and copper have been reported to be between 1.13 to 2.4%, 1.26 to 2.1%, and 0.6 to 4%, respectively. Inter-assay CVs are between 3.7 to 4.1 for iron, 3.9 to 9.7 for copper, and 1.6 to 5% for magnesium.^24^ The hair samples were cleaned with 10% nitric acid and rinsed in water for 10 minutes. The samples were dried before weighing. The samples were digested in 15 mL glass tubes with 0.5 mL of extra pure nitric acid 65% (Merck, No 100443). An 80°C bain-marie was used to heat samples for 120-180 minutes. The samples were diluted to 10 mL with high-purity water (approximately 5% v/v nitric acid). Standards were prepared in acid-matched (~5% v/v nitric acid) solutions. The reporting and analytical methodology in this study were carried out by Kimiazi Analysis Research Lab.

###  Statistical Analysis

 Baseline demographic characteristics were compared between case and control participants with Kruskal-Wallis and chi-square tests. The potential predictors of hair mineral concentrations were assessed using partial correlations.

We assessed the relationship between each mineral and ESCC risk using multivariable logistic regression models. We identified potential risk factors for ESCC based on the existing literature, particularly the previous studies in the GCS,^19,21^ which included factors such as age (years), sex, place of residence (urban, rural), smoking (never, ever), wealth score (low, medium, high), opiate use (never, ever), body mass index ( < 18.5, ≥ 18.5, ≥ 25, ≥ 30), education (no formal, formal education), ethnicity (non-Turkmen, Turkmen), physical activity (irregular non-intense, regular non-intense, irregular or regular intense),^25,26^ and fruit and vegetable intake (g/d). Based on appliance ownership, wealth score was calculated as a proxy for socioeconomic status.^27^ Certain risk factors were found to be linked to hair mineral levels in the partial correlation squared analysis (Supplementary file 1, Table S1). Nevertheless, in our report, we presented both unadjusted (crude) and fully adjusted models to account for potential confounding variables and provide a comprehensive understanding of the associations. Conditional and unconditional models were used. However, the unconditional models offered more precise estimates; thus, we reported the results of unconditional models throughout the paper. Mineral concentrations were categorized into tertiles according to concentrations of each mineral among controls. For the linear trend test, we used the median of tertiles. The log-transformed values were used in continuous analyses.

We used the Stata software (version 16, Stata Corp, College Station, TX, USA). We considered *P* values < 0.05 as statistically significant.

## Results

During follow-up (median: 7.2 years and interquartile range: 6.4–8.0 years), 207 participants with ESCC were identified. From these 207 ESCC cases, 96 participants who had enough hair samples (more than 20 mg) were selected for inclusion in the analysis. These 96 ESCC cases were age-matched 1:1 with controls ( + /- 1 year). The demographic information of participants is shown in Table 1.

**Table 1:** Baseline Characteristics of Incident Cases of Esophageal Squamous Cell Carcinoma and Controls in the Golestan Cohort Study

**Characteristics**	**Case Subjects**	**Control Subjects**	***P*** **Value**^a^
Age, years, mean ± SD	57.7 ± 1.0	57.0 ± 1.0	0.61
Male, No. (%)	22 (22.9)	18 (20.4)^b^	0.69
BMI, kg/m, mean ± SD	23.8 ± 0.4	27.4 ± 0.5	< 0.001
Smoker, No. (%)	13 (13.5)	9 (10.2)	0.49
Wealth score, No. (%)			
Low	66 (68.7)	27 (30.7)	< 0.001
Medium	16 (16.7)	25 (28.4)	
High	14 (14.6)	36 (40.9)	
Physical activity score, mean ± SD	1.2 ± 0.0	1.3 ± 0.6	0.68
Place of residence, rural, No. (%)	89 (92.7)	68 (77.3)	0.003
Ethnicity, Turkmen, No. (%)	79 (82.3)	72 (82)	0.93
Opium user, No. (%)	15 (15.6)	14 (15.9)	0.95
Education, no formal education, No. (%)	83 (86)	73 (82.9)	0.51
Fruit intake, g/d, mean ± SD	123.4 ± 108.4	156.9 ± 123.0	0.04
Vegetable intake, g/d, mean ± SD	105.2 ± 6.1	120.8 ± 6.8	0.09
Baseline hair mineral levels (ppm)^b^, median (IQR)			
Iron	35.4 (17.8-54.5)	25.8 (11.6 -44.0)	0.02
Copper	19.3 (4.8- 40.0)	18.3 (9.6-42.4)	0.42
Magnesium	41. 7 (28.9-65.2)	50.0 (33.9-68.0)	0.07

^a^* P* values come from the Kruskal-Wallis test. ^b^ Measured using ICP-Mass.

ESCC cases had higher baseline iron concentrations in their hair. As Table 1 shows, the concentrations of magnesium and copper were not significantly different between cases and controls. Table S1 shows the results of squared partial correlations of iron, copper, and magnesium to demonstrate the relation between mineral concentrations and each potential risk factor of ESCC.

Iron concentration in hair collected at baseline was positively related to the risk of ESCC, in continuous analyses (OR = 1.41, 95% CI = 1.03-1.92). However, in tertiles analysis, comparing the highest tertiles of hair iron concentration to the reference, no statistically significant association was found (OR_T3 vs. T1_ = 1.81, 95% CI = 0.77-4.28) (Table 2).

**Table 2:** Odds Ratios of Esophageal Squamous Cell Carcinoma, for Hair Mineral Concentrations in the Golestan Cohort Study

	**T1**	**T2**	**T3**	***P*** ** for trend**^a^	**Continuous **
Iron					
No of cases	24	28	43		
OR^b^(95% CI)	1	1.17 (0.55-2.45)	1.79 (0.88-3.65)	0.09	1.40 (1.07-1.82)
OR^c^ (95% CI)	1	1.00 (0.41-2.42)	1.81 (0.77-4.28)	0.16	1.41 (1.03-1.92)
Copper					
No of cases	25	37	33		
OR^b^(95% CI)	1	1.48 (0.72-3.03)	1.32 (0.64-2.73)	0.62	1.06 (0.90-1.26)
OR^c^ (95% CI)	1	1.62 (0.67-3.92)	2.56 (1.01-6.54)	0.06	1.21 (0.97-1.51)
Magnesium					
No of cases	43	26	26		
OR^b^(95% CI)	1	0.58 (0.29-1.17)	0.67 (0.33-1.35)	0.29	0.60 (0.36-1.00)
OR^c^ (95% CI)	1	0.55 (0.24-1.26)	0.75 (0.32-1.78)	0.54	0.58 (0.31-1.10)

^a^ *P* values for the trend were calculated by using the median value of each tertile in the model. ^b^ Crude model; OR (95% CI) were calculated by using unconditional logistic regression models. ^c^ Adjusted for age (years), sex, place of residence (urban, rural), smoking (never, ever), wealth score (low, medium, high), opiate use (never, ever), body mass index ( < 18.5, ≥ 18.5, ≥ 25, ≥ 30), education (no formal, formal education), ethnicity (non-Turkmen, Turkmen), physical activity (irregular non-intense, regular non-intense, irregular or regular intense), and fruit and vegetable intake (g/d); ORs (95% CI) were calculated using unconditional logistic regression models.

Participants in the third tertile of hair copper had a higher risk of ESCC, but it was not statistically significant (OR_T3 vs. T1_ = 2.56, 95% CI = 1.00-6.54). Also, in models using the continuous (log) hair copper concentrations, no statistically significant relationship with the risk of ESCC was noted (OR = 1.21, 95% CI = 0.97-1.51) (Table 2).

Higher hair magnesium concentration was associated with a decreased risk of ESCC, but it was not statistically significant in either the tertiles test (OR_T3 vs. T1_ = 0.75, 95% CI = 0.32-1.78) or the continuous test (OR = 0.58, 95% CI = 0.31-1.10) (Table 2).

 Adjustment for hair mass did not change any of the results significantly. Interaction tests for age and sex were not significant for any of the measured minerals, according to the Wald test.

## Discussion

 In this cohort study, using hair samples to measure mineral status, higher hair iron concentration was related to a higher risk of subsequent ESCC. Copper and magnesium concentrations in hair samples were not related to the risk of developing ESCC.

Our findings are consistent with the results of a study in the Netherlands Cohort, in which a higher dietary intake of heme iron, as reflected in the FFQ, was related to a higher risk of developing ESCC.^9^ Conversely, Cross et al also used data extracted from an FFQ and found no statistically significant relation between dietary heme iron and the risk of ESCC.^10^ The inconsistency could be due to changes in heme iron during different cooking methods,^28^ or measurement errors due to the use of a FFQ. The comparison between hair iron and iron intake may not be entirely warranted because nitric oxide from the diet or drinking water can directly interact with heme proteins, resulting in the formation of N-nitroso compounds, which are known carcinogens.^29^ Our use of a biomarker, hair mineral concentration, to assess the iron status, avoids some of the measurement error inherent in questionnaire-based data collection. Abnet et al assessed mineral status using esophageal biopsy specimens in 60 ESCC cases and 72 dysplasia controls from a Nutrition Intervention Trial in China and found no relationship between iron concentration in biopsy samples of esophagus and risk of ESCC in subjects with dysplasia.^30^ Normal, non-dysplastic tissue was not available for this study and this comparison between the iron concentration in tumor tissue and pre-cancerous tissue may have attenuated the association. There is biological plausibility for our results. Tumors demand extra iron for their rapid growth.^6^ High iron concentrations can produce reactive oxygen species, that could damage DNA and develop gene mutation.^6^

There was no significant relationship between copper status and the risk of developing ESCC in our study. Case-control studies have demonstrated conflicting findings.^31,32^ In our previous analyses, using FFQ in the Golestan Cohort Study, we found no linear association between copper intake and developing ESCC.^25^ Abnet et al reported that copper concentrations in esophageal biopsy samples were not related to the risk of developing ESCC,^30^ which is consistent with our results.

We found no significant association between magnesium and ESCC in the Golestan Cohort Study. However, there is a nonlinear U-shaped association between dietary intake of magnesium and the risk of developing ESCC.^25^ The discrepancy between the results of mineral status based on FFQ and biomarkers has been reported in previous studies,^33,34^ because of measurement errors or various metabolisms in various participants.

The strengths of the current study are the prospective design of the study, nested within a cohort study with an excellent follow-up rate. We also used a long-term exposure, non-invasive, and easy-to-store biomarker. Our study has some limitations. First, our sample size was small. The lack of significant relationships among variables appears to be related to the sample size of the study. It is the first study of an association between hair mineral concentrations and the risk of developing ESCC in a prospective cohort study and should be confirmed in future studies. Hair mineral concentrations may vary with hair growth rate, hair color, and other cosmetic products,^35^ and we did not have this information about our subjects.

## Conclusion

 In conclusion, we observed that higher hair iron concentrations were associated with an increased risk of ESCC. Since we are reporting an association between hair minerals and the risk of developing ESCC for the first time in a prospective cohort study, it needs to be replicated in future studies. Hair samples, which are collected non-invasively and are easy to store, could be used to estimate mineral status in future studies.

## Supplementary Files


Supplementary file 1 contains Table S1.
