# Supplementary material for: Iron, Copper, and Magnesium Concentration in Hair and Risk of Esophageal Cancer: A Nested Case-Control Study
Source: Arch Iran Med. 2023 Dec 1;26(12):665–70. doi: 10.34172/aim.2023.98 (PMC10915918; doi:10.34172/aim.2023.98)
Supplement: Supplementary file 1 — contains Table S1. [file aim-26-665-s001.pdf]

Arch Iran Med. December 2023;26(12):665-S1

10.34172/aim.2023.98

Supplementary file 1

Table S1. Correlations between hair mineral concentrations and suspected risk factors for esophageal squamous cell carcinoma in the controls from the Golestan Cohort Study.

	**Partial correlation squared (percent)**		
**Variable**	**Iron**	**Copper**	**Magnesium**
Sex	7.8*	0.2	4.8
Age	0.5	0.5	0.5
Education	3.8	0.1	0.5
BMI, kg/m^2	0.6	0.7	0.3
Place of residence	0.9	3.8	4.0
Opium	0.1	0.6	0.1
Smoking	9.2*	0.0	0.2
Physical activity	7.6*	0.6	5.2
Vegetable intake	0.0	0.6	0.0
Fruit intake	0.4	1.3	0.2
Ethnicity	1.9	1.8	0.6
Wealth score	1.1	0.3	0.0
Other minerals			
Iron	-	0.2	2.1
Copper	0.2	-	4.8*
Magnesium	2.1	4.8*	-

*P<0.05
